# Influence of muscle volume on jumping performance in healthy male and female youth and young adults

**DOI:** 10.1186/s13102-023-00639-x

**Published:** 2023-03-06

**Authors:** Souhail Bchini, Nadhir Hammami, Taoufik Selmi, Dalenda Zalleg, Anissa Bouassida

**Affiliations:** 1 High Institute of Sport and Education of Kef, University of Jendouba, Kef, Tunisia; 2 Higher Institute of Sport and Physical Education of Ksar Said, University of “La Manouba”, Manouba, Tunisia

**Keywords:** Age, Sex, Leg length, Muscle volume, Vertical jump

## Abstract

**Background:**

Sex differences that appear throughout puberty have a substantial impact on the training process. It remains unclear what effect these sex differences should have on how training programs are planned and performed and what objectives should be established for boys and girls of different ages**.** This study aimed to investigate the relationship between vertical jump performance and muscle volume based on age and sex.

**Methods:**

One hundred eighty healthy males (n = 90) and females (n = 90) performed three different types of vertical jumps (VJ): squat jump (SJ), counter movement jump (CMJ), and counter movement jump with arms (CMJ with arms). We used the anthropometric method to measure muscle volume.

**Results:**

Muscle volume differed across age groups. There were significant effects of age, sex, and their interaction on the SJ, CMJ, and CMJ with arms heights. From the age of 14–15, males exhibited better performances than females, and large effect sizes became apparent in the SJ (d = 1.09, P = 0.04), CMJ (d = 2.18; P = 0.001) and CMJ with arms (d = 1.94; P = 0.004). For the 20–22-year-old age group, there was a significant difference in VJ performance between males and females. Extremely large effect sizes became apparent in the SJ (d = 4.44; P = 0.001), CMJ (d = 4.12; P = 0.001) and CMJ with arms (d = 5.16; P = 0.001). When performances were normalized to the lower limb length, these differences persisted. After normalization to muscle volume, males exhibited better performance when compared to females. This difference persisted only for the 20–22-year-old group on the SJ (p = 0.005), CMJ (p = 0.022) and CMJ with arms (p = 0.016). Among male participants, muscle volume was significantly correlated with SJ (r = 0.70; p < 0.01), CMJ (r = 0.70; p < 0.01) and CMJ with arms (r = 0.55; p < 0.01).

**Conclusions:**

The results indicate that muscle volume may be one of the major determining factors in sex differences in vertical jumping performance.

**Supplementary Information:**

The online version contains supplementary material available at 10.1186/s13102-023-00639-x.

## Introduction

It has been reported that during postpuberty, morphotypes for both sexes have a noticeable effect on physical performance, such that males tend to exhibit better performances than females [1]. Moreover, during puberty, hormonal differences cause a significant increase in fat mass among female participants and a significant increase in muscle mass among male participants [2]. Accordingly, among females, higher fat infiltration and lower muscle mass may explain why they tend to exhibit worse physical performances than males [1]. Specifically, compared to males, females have a greater intramuscular fat content [3] and greater amount of connective tissue [4]. When expressed in terms of body mass, no differences in the lower limb anaerobic power was observed between sexes; however, differences in upper limbs anaerobic power were observed [5]. Regarding lower limbs, Croix et al. [6] showed that in young participants aged 10–12 years, thigh muscle volume had a positive impact on muscle power during the Wingate test. Lower limb strength, explosiveness and coordination have been reported to be essential factors that positively affect jumping performance [7], which is considered to be a basic function parameter that is used to predict and assess physical performance as well as to identify talent [8, 9]. Previous studies have shown that vertical jump performance is related to many factors, such as sex, sports specialization, and accident risk [10–14], and is affected by physiological and biomechanical factors [15]. Several researchers [16–18] have conducted studies with anthropometric variables. Roschel et al. [16] found a significant negative correlation between the sum of skinfold thickness and vertical jump among karate athletes. In addition, a negative correlation has been observed between recreational athletes' body fat percentage and jump height [18]. Furthermore, Markovic [19] investigated the correlation between body mass and vertical jump height and showed that body mass was independent of jump height. Davis et al. [18] observed no significant correlation between body height and vertical jump. Aslan et al. [17] demonstrated that among sub-elite athletes, body height has no significant impact on vertical jump.

Various studies have examined the impact of different variables on anaerobic performance, including age, sex, muscle type, mass, cross-section, hereditary traits, training, and body composition [20, 21]. Additionally, the development of muscle strength in anaerobic sports is greatly influenced by muscle fascicle length, leg volume, and muscle mass [22]. As a result, for improved anaerobic performance, athletes need to have higher amounts of muscle mass, muscle cross section, and leg volume and mass [23]. The total muscle volumes of the leg and thigh as estimated by anthropometry are positively correlated with sprint velocity, squat jump (SJ) height, and absolute leg force [24, 25]. In addition, lower limb muscle volume are determining factor in muscle power for both sexes [26]. Jumping performance increases during growth, with sex differences manifesting from 14 years onwards due to the much greater increase in leg length and LMV among boys than among girls between 11 and 16 years of age [27]. To the best of the present authors' knowledge, no previous research has examined the correlation between muscle volume and vertical jump performance across sex and age groups during the period from prepuberty to adulthood.

This study aimed to determine the relationship between vertical jump and muscle volume based on sex and age. We hypothesized that the differences in vertical jumping performance between sexes could be explained by differences in muscle volume.

## Materials and methods

### Participants

One hundred eighty students participated in this study, including ninety healthy males (mean ± SD: age: 15.11 ± 4.79 years, height: 161.4 ± 17.13 cm, body mass: 53.14 ± 17.79 kg) and ninety healthy females (mean ± SD: age: 15 ± 5 years, height: 155.31 ± 12.28 cm, body mass: 49.22 ± 14.11 kg). Participants were from different age groups, including before puberty (i.e., 9 to 10 years; n = 30 male and 30 females), puberty (i.e., 14 to 15 years; n = 30 males and 30 females) and adulthood (20 to 22 years; n = 30 male and 30 females). A general health questionnaire was completed by participants and did not present any medical restrictions. Individuals aged 9–10 years old, 14–15 years old, and 20–22 years old participated in two hours of physical education per week at school or university. All participants who engaged in other physical activity or sports were eliminated from this study. Puberty was assessed and verified according to the Tanner model [28], which was used for inclusion or exclusion between groups. After a detailed presentation of the investigation's objectives, advantages, and potential risks, all participants and their parents provided written informed consent. The study was conducted according to the Declaration of Helsinki for human experimentation. The local research ethics committee of the High Institute of Sport and Physical Education of Kef, University of Jendouba, approved the protocol with the code number a10-2019, authorized on January 25, 2019.

### Design

The study was conducted from March to April 2018. Vertical jump performance was expressed in centimetres (cm). Each participant performed 3 trials for SJ, CMJ and CMJ with arms, and the highest jump height for each type of jump was used for further analysis. Each trial was recorded using an Optojump (Optojump, Microgate, Bolzano, Italy). The lower limb muscle volume was estimated using the anthropometric method [29].

### Procedures

Two weeks before the beginning of the experiment, participants were familiarized with the tests, and all testing sessions were conducted at the same time of day to avoid any diurnal variation in performance [30]. At the first visit, anthropometric measurements (standing and sitting height, leg length, body mass and muscle volume) were evaluated and were used as a familiarization session during which the participants received instructions to correctly perform the three modalities of jump. They were required to practice between 5 and 10 maximal jumps for each modality of jump. During the second visit, vertical jumping performances were assessed using the SJ, CMJ and CMJ with arms. Experimental conditions were completed in three weeks. All participants performed three trials for each jump. The jumps were separated by a 2-min rest to ensure sufficient recovery. All tests were performed with the same verbal encouragement to ensure optimal performance by athletes. Before each experimental condition, 15 min of standardized warm-up was performed consisting of jogging and dynamic stretching, after which 3 min of passive rest was taken and measurements were taken.

### Anthropometric measurements

Anthropometric data were assessed, including standing body height, leg length, body mass (BM), and body fat percentage (% fat). All measurements were taken by the same person three times using techniques established by the international biological program [31]. Circumferences and skin-fold thickness at different levels of the thigh and the calf, the length of the lower, and the femoral condyles breadth are measured to estimate the muscle volume of the lower limbs [29]. Muscle volumes were estimated in accordance with Eq. 1

1 $${\text{Muscle}}\;{\text{volume}}\, = \,{\text{total}}\;{\text{limb}}\;{\text{volume}}\, - \,\left( {{\text{fat}}\;{\text{volume}}\, + \,{\text{bone}}\;{\text{volume}}} \right)$$

The total limb volume was estimated as the volume of a cylinder, based on its length (L), corresponding to the distance from the trochanter major to the lateral malleolus for the lower limb, and the mean of five limb circumferences lower limbs (for the maximal thigh, mid-thigh, just below the patella, maximal calf and just above the ankle) in accordance with Eq. 2

2 $${\text{Total}}\;{\text{limb}}\;{\text{volume}}\, = \,\left( {\sum {\text{C2}}} \right) \cdot {\text{L}}/{62}.{8}$$

where ∑C2 is the sum of the squares of the five circumferences of the corresponding limb. Skin folds were assessed using a standard Harpenden calliper (Baty International, Burgess Hill, Sussex, UK). The fat volume was calculated using Eq. 3

3 $$\left( {\sum {\text{C}}/{5}} \right) \cdot \left( {\sum {\text{S}}/{\text{2n}}} \right){\text{ L}}$$

where ∑S is the sum of four skinfolds for the lower limb (front of mid-thigh, back of mid-thigh, back of calf and outside of calf) and ''n'' represents the number of skin folds measured.

Bone volume was calculated as

4 $$\pi \cdot \left( {{\text{F}} \cdot {\text{D}}} \right){2} \cdot {\text{L}}$$

where D is the femoral intercondylar diameter, F is a geometric factor (0.235 for the lower limb), and L is the limb length as measured above.

Bone volume was calculated as π ∙ (F ∙ D) 2∙ L, where D is the femoral intercondylar diameter, F is a geometric factor (0.235 for the lower limb), and L is the limb length as measured above.

### Vertical jumps

Vertical jump height was assessed using the SJ, CMJ and CMJ with arms. For the SJ, participants started from the upright standing position with their hands on their hips. They were then instructed to flex their knees and hold a predetermined knee position (~ 90°) for 3 s. At this moment, participants were instructed to jump as high as possible without performing any countermovement phase. In the CMJ, participants started from the upright standing position with their hands on their hips. They were then instructed to flex their knees (~ 90°) as quickly as possible and then jump as high as possible in the ensuing concentric phase. In the CMJ with arms, participants were instructed to perform a CMJ with arm swing during the execution of the jump (i.e., hands were free to move). For jumping tests, participants were asked to jump as high as possible and to land in the same place that they jumped from to avoid movement in any direction [32]. To ensure that the participants were using their leg extensors, they were asked to keep their torso upright [33]. The performance was recorded as the height of the jump using an Optojump (Optojump, Microgate, Bolzano, Italy), which consists of 2 parallel bars placed approximately 1 m apart and parallel to each other. Optojump bars were connected to a portable computer, and the propriety software (software, version 3.01.0001) allowed jump height quantification. The optical system transmits infrared light 1–2 mm above the floor. When the light is interrupted by the feet. The unit triggers a timer within 1 ms, which allows the measurement of flight time [34]. The jump height (H) expressed in centimetres was calculated with the following formula

5 $${\text{H}}\, = \,{\text{g}}\, \times \,{\text{t2}}/{8}$$

where H is the vertical jump (cm), t is the flight time (s) and g is the acceleration due to gravity (9.81 m·s^-2^). The best result was kept for the analysis, and there was a 45-s recovery time between trials (Additional file 1).

### Statistical analyses

The mean and standard deviation are used to present the data. The statistical analysis was performed using SPSS 18.0 statistical software (IBM Corp. Armonk, NY, USA). The normality of the datasets was checked and confirmed using the Kolmogorov–Smirnov test. Sphericity was tested and confirmed using the Mauchly test. Data were analysed using a two-way analysis of variance (age group × sex) with repeated measurements to compare experimental conditions, and the Scheffé test was used as a post hoc test. Lower limb length and muscle volume were inserted as covariables to control their effects. Comparisons between males and females for each variable were made using independent t tests. The reliability of the vertical jumping tests was assessed by calculating the intraclass correlation coefficients (ICCs) and their 95% confidence intervals. We used one-way ANOVA to obtain the ICC of repeated interval scale measures [35]. As a rule, an ICC of.0.90 is considered high for physiological field tests [36].

The effect size (Cohen's d) analysis was used to determine the size of the differences between variables [37]. Cohen’s d was interpreted using the following thresholds: < 0.20 (trivial); 0.20–0.60 (small); 0.60–1.20 (moderate); 1.20–2.0 (large); 2.0–4.0 (very large); and > 4.0 (extremely large) [37]. Moreover, upper and lower 95% confidence intervals of the difference (95% CIds) were calculated for corresponding variation. The relationship between lower limb length and muscle volume and jumping performance was evaluated using Pearson product correlations. Statistical significance was set at p < 0.05.

## Results

### Anthropometric characteristics according to age and sex

The anthropometric characteristics for different age and sex groups are presented in Table 1. For the group aged 9 to 10 years old, males showed higher lower limb muscle volume than females (95% CId = 3.52 ± 3.89; d = 0.66, P = 0.038). Males had higher lower limb muscle volumes than females in the 14- to 15-year-old group (95% CId = 5.87 ± 6.23; d = 2.05, P = 0.001) and in the 20- to 22-year-old group (95% CId = 8.2 ± 8.57; d = 3.11, P = 0.001).

**Table 1:** Anthropometric characteristics of participants according to their age group (9–10 years old; 14–15 years old; and 20–22 years old) and their sex

		Body mass (kg)	Body fat percentage (%)	Height (cm)	Sitting height (cm)	LLL: Lower limb length (cm)	MV:Muscle volume (L)
9–10 years	Males	34.63 ± 7.26	19.11 ± 2.89	139.67 ± 8.07	72.30 ± 4.47	67.37 ± 5.06	3.71 ± 0.53
	Females	33.96 ± 7.82	16.99 ± 5.67	140.93 ± 6.98	72.63 ± 4.60	68.30 ± 5.53	3.26 ± 0.40
	Dif %	− 1.97	− 12.48	+ 0.89	+ 0.45	+ 1.46	− 13.80*
14–15 years	Males	55.66 ± 12.27	11.48 ± 5.54	169.43 ± 7.59	81.70 ± 4.09	87.77 ± 4.92	6.05 ± 0.84
	Females	54.27 ± 8.19	22.67 ± 5.56	161.80 ± 6.27	81.80 ± 4.21	79.10 ± 6.81	4.57 ± 0.19
	Dif %	− 2.56	+ 49.36***	− 4.71***	+ 0.12	− 10.96***	− 32.38***
20–22 years (young adults)	Males	69.13 ± 11.95	18.59 ± 7.53	175.10 ± 5.28	86 ± 5.16	89.27 ± 6.08	8.38 ± 0.51
	Females	59.44 ± 10.37	26.81 ± 4.96	163.20 ± 7.26	77.63 ± 7.18	85.73 ± 6.62	6.36 ± 0.33
	Dif %	− 16.30***	+ 30.66***	-7.29***	-10.65***	-4.71	− 31.76***

Data are presented as the means ± SD. Dif %: differences between male and female participants of the same age group expressed in female participant value percentage. LLL: lower limb length (in cm); MV: lower limb muscle volume in litre (L); * (P < 0. 05) and *** (P < 0.001): significant differences between both sexes for each age group

### Vertical jumping performance

Table 2 presents the ICC values to assess the reliability of vertical jumping performance. The ICC for SJ height for males and females was 0.92, for the CMJ it was 0.95 and for CMJ with arms it was 0.95.

**Table 2:** Intraclass correlation coefficients for the relative reliability of jumping vertical performance

	VJ	ICC	95% CI
Males	SJ	0.921	0.900–0.922
	CMJ	0.952	0.950–0.953
	CMJ arms	0.954	0.950–0.956
Females	SJ	0.920	0.919–0.921
	CMJ	0.954	0.952–0.954
	CMJ arms	0.957	0.955–0.957

ICC = intraclass correlation coefficient; CI = confidence interval, VJ = vertical jump

Vertical jump performances for males and females from different age groups are presented in Table 3. A significant age effect was found for the SJ (F_(2.172)_ = 5.49, P = 0.005), CMJ (F_(2.172)_ = 5.68, P = 0.004) and CMJ with arms (F_(2.172)_ = 3.89, P = 0.02). The post hoc Scheffé test revealed that a difference occurred for both sexes between the 9–10 years and 14–15 years groups (P = 0.032) and between the 14–15 years and 20–22 years groups (P = 0.001). Significant sex differences were observed between males and females for SJ (F_(1.172)_ = 26.40; P = 0.002), CMJ (F_(1.172)_ = 8.87; P = 0.003) and CMJ with arms (F_(1.172)_ = 17.03; P = 0.001). An interaction effect was observed for SJ (F_(2.172)_ = 14.23; P = 0.001), CMJ (F_(2.172)_ = 10.27; P = 0.001) and CMJ with arms (F_(2.172)_ = 6.85; P = 0.002).

**Table 3:** High jump performances realized in vertical jump by participants according to their age group (9–10 years old; 14–15 years old; and 20–22 years old) and their sex

		SJ	CMJ	CMJ with arms
9–10 years	Males	14.59 ± 5.19	16.61 ± 3.80	19.37 ± 5.82
	Females	13.47 ± 4.11	15.15 ± 2.78	16.36 ± 4.05
	Dif %	− 8.31	− 9.64	− 18.40*
	Effect size (Cohen’s d)	0.51(small)	0.80 (moderate)	1.35 (large)
	P Value	0.36	0.095	0.024*
14-15 years	Males	18.40 ± 3.53	20.78 ± 3.53	22.65 ± 6.09
	Females	16.15 ± 4.95	16.64 ± 2.88	17.83 ± 6.20
	Dif %	− 13.93*	− 24.88***	− 27.03***
	Effect size (Cohen’s d)	1.09 (moderate)	2.18 (large)	1.94 (large)
	P Value	0.04*	0.001***	0.004***
20-22 years (young adults)	Males	28.33 ± 6.23	29.87 ± 8.89	31.81 ± 8.35
	Females	18.07 ± 4.49	19.10 ± 4.70	20.05 ± 5.86
	Dif %	− 56.78***	− 56.39***	− 58.65***
	Effect size (Cohen’s d)	4.44 (extremely large)	4.12(extremely large)	5.16(extremely large)
	P Value	0.001***	0.001***	0.001***

Dif %: differences between male and female participants of the same age group expressed in female participant value percentage.SJ: squat jump height (in cm).CMJ: counter movement jump height (in cm).CMJ arm: Counter movement jump height with arms (in cm). * (P < 0. 05) and *** (P < 0.001): significant differences between both sexes for each age group

At the ages of 9–10 years old, there was no significant difference between males and females in SJ and CMJ, but there was a difference in CMJ with arms. Small effect sizes became apparent in SJ (d = 0.51; P = 0.36), moderate in CMJ (d = 0.80; P = 0.095) and large effect sizes in CMJ with arms (d = 1.35; P = 0.024).

At the ages of 14–15 years old, males performed significantly better than females, and a moderate effect size became apparent in performance with a large effect size in SJ (d = 1.09, P = 0.04), CMJ (d = 2.18; P = 0.001) and CMJ with arms (d = 1.94; P = 0.004). Males aged 20 to 22 years old performed significantly better than females, and an extremely large size effect became apparent in SJ (d = 4.44; P = 0.001), CMJ (d = 4.12; P = 0.001) and CMJ with arms (d = 5.16; P = 0.001). These differences were also maintained when performances were normalized for lower limb length. Even after normalization to lower limbs muscle volume, males exhibited better performance than females in the 20- to 22-year-old age group in SJ (P = 0.005), CMJ (P = 0.022) and CMJ with arms (P = 0.016) (Table 4).

**Table 4:** Performances realized through vertical jump by participants according to their age group (9–10 years old; 14–15 years old; and 20–22 years old) and their sex with reference to muscle volume and lower limb length

		SJ/MV	SJ/LLL	CMJ/MV	CMJ/LLL	CMJ with arms/MV	CMJ with arms/LLL
9–10 years	Males	3.90 ± 1.47	0.22 ± 0.08	4.55 ± 1.72	0.25 ± 0.06	5.26 ± 1.61	0.29 ± 0.08
	Females	4.14 ± 1.17	0.20 ± 0.06	4.70 ± 0.96	0.22 ± 0.04	5.03 ± 1.14	0.24 ± 0.06
	Dif %	+ 5.79	− 10	+ 3.19	− 13.64	− 4.57	− 20.83
	P Value	NS	NS	NS	NS	NS	0.01*
14-15 years	Males	3.11 ± 0.75	0.21 ± 0.04	3.54 ± 1.14	0.24 ± 0.05	3.86 ± 1.31	0.26 ± 0.07
	Females	3.53 ± 1.06	0.21 ± 0.07	3.64 ± 0.60	0.21 ± 0.04	3.90 ± 1.35	0.23 ± 0.08
	Dif %	+ 11.90		+ 2.75	− 14.29*	+ 1.03	− 13.04
	P Value	0.02*	NS	0.05*	0.01*	NS	0.01*
20-22 years (young adults)	Males	3.39 ± 0.79	0.32 ± 0.06	3.58 ± 1.08	0.33 ± 0.09	3.81 ± 1.03	0.33 ± 0.09
	Females	2.84 ± 0.69	0.21 ± 0.06	3.01 ± 0.77	0.22 ± 0.06	3.17 ± 0.96	0.24 ± 0.07
	Dif %	− 19.37	− 52.38	− 18.94	− 50	− 20.19	− 43.48
	P Value	0.005***	0.001***	0.02*	0.001***	0.001***	0.001***

Data are presented as the means ± SD. Dif %: differences between male and female participants of the same age group expressed in female participant value percentage. SJ: squat jump height (in cm); CMJ: counter movement jump height (in cm); CMJ arm: Counter movement jump height with arms (in cm); LLL lower limb length (in cm); MV: lower limb muscle volume in litre (l). For convenience, the report of SJ/MV, CMJ/MV and CMJ with arm/MV is presented and discussed in the text without unit. * (P < 0. 05) and *** (P < 0.001): significant differences between both sexes for each age group

### Relationships between vertical jump performance and anthropometric characteristics

**Fig. 1:**
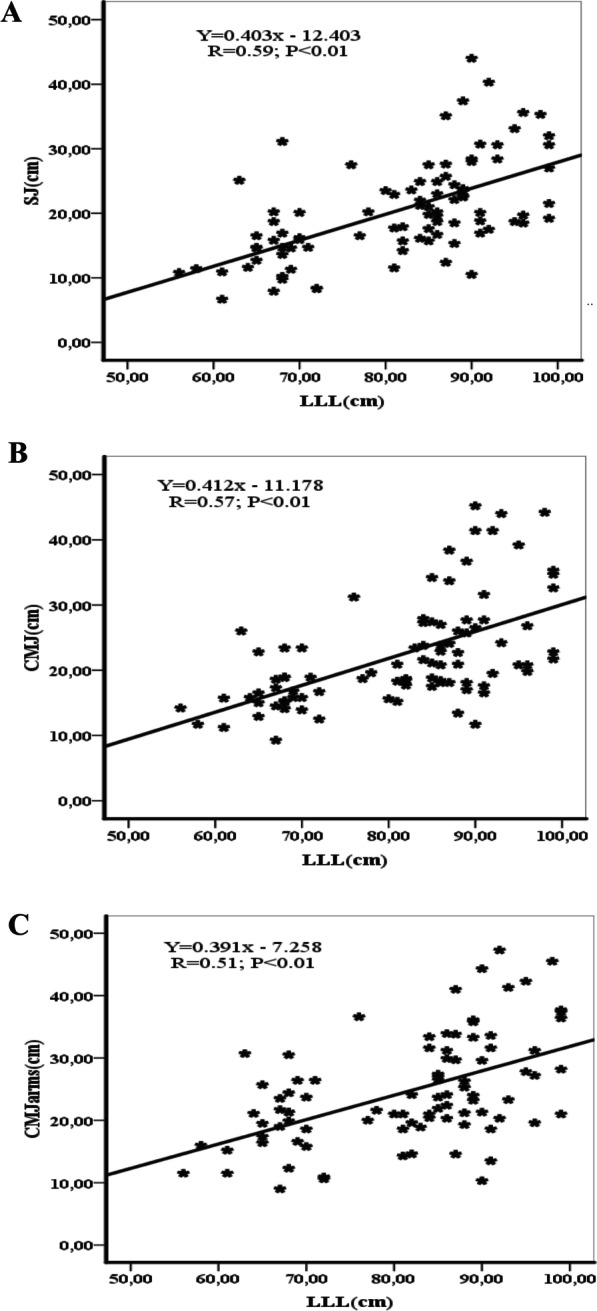
Correlation coefficients (r) between lower limbs length and jumping performance for males. LLL = lower limbs length, SJ = squat jump, CMJ = counter movement jump, CMJ with arms = counter movement jump with arms. *(p < 0.05), ** (p < 0.01)

**Fig. 2:**
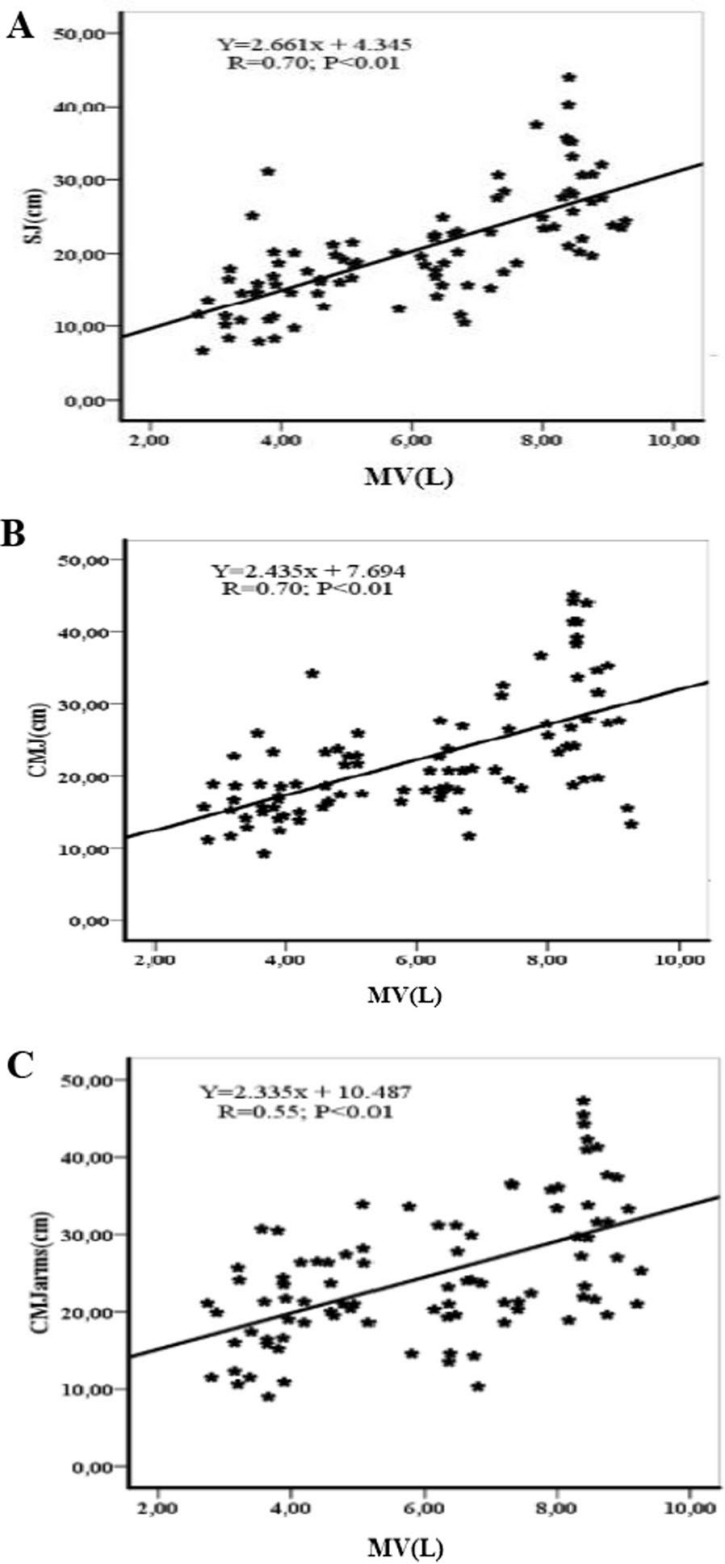
Correlation coefficients (r) between muscle volume (MV) and jumping performance for males. MV = muscle volume, SJ = squat jump, CMJ = counter movement jump, CMJ with arms = counter movement jump with arms. *(p < 0.05), ** (p < 0.01)

**Fig. 3:**
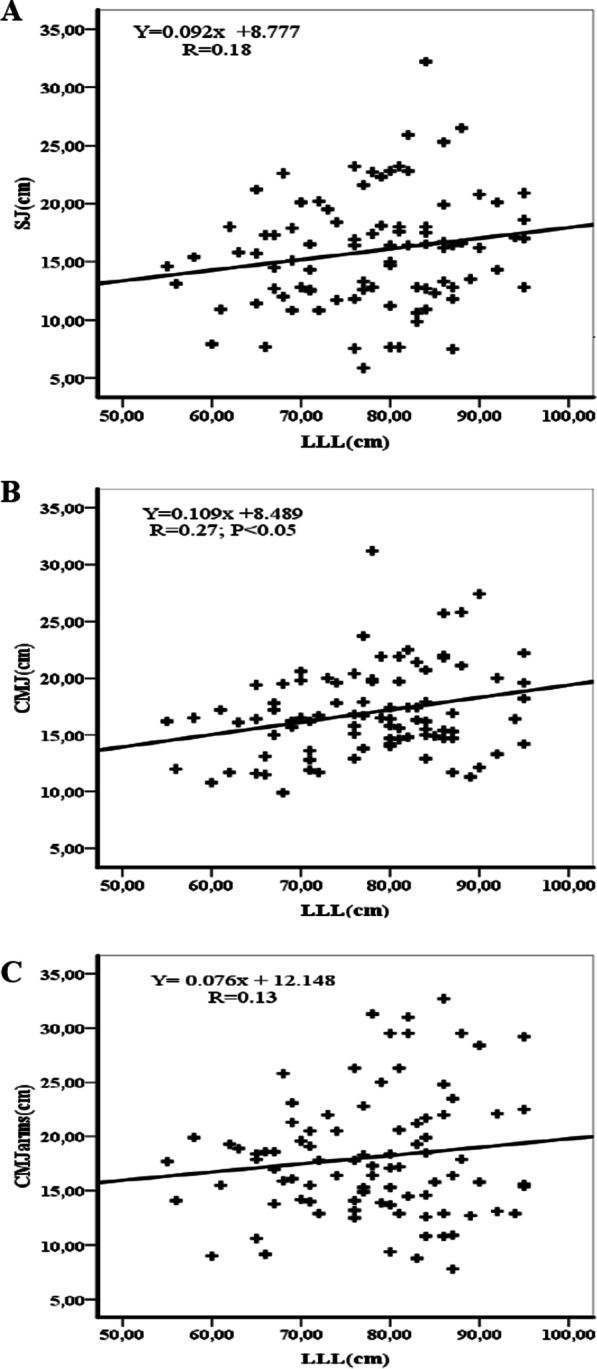
Correlation coefficients (r) between lower limbs length and jumping performance for females. LLL = lower limb length, SJ = squat jump, CMJ = counter movement jump, CMJ with arms = counter movement jump with arms. *(p < 0.05), ** (p < 0.01)

**Fig. 4:**
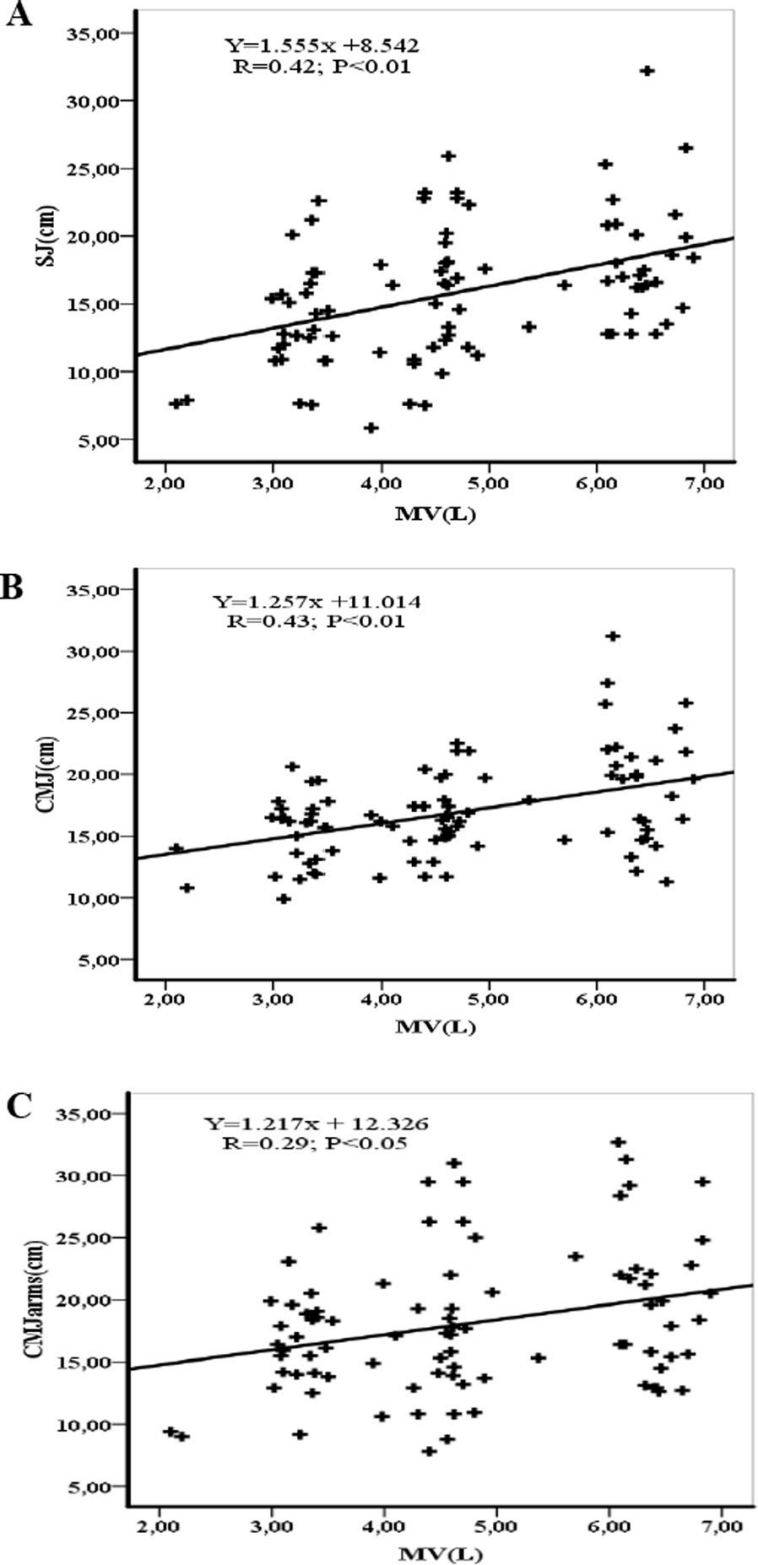
Correlation coefficients (r) between muscle volume (MV) and jumping performance for females. MV = muscle volume, SJ = squat jump, CMJ = counter movement jump, CMJ with arms = counter movement jump with arms. *(p < 0.05), ** (p < 0.01)

Figures 1, 2, 3 and 4 show the correlations between anthropometric parameters (lower limb length, muscle volume) and jumping performance. There was a significant correlation between lower limb length (LLL) and jumping performance for males (Fig. 1). There was a significant correlation between MV and SJ for males (r = 0.70; p < 0.01) (Fig. 2A) and females (r = 0.42; p < 0.01) (Fig. 4A), a significant correlation between MV and CMJ for males (r = 0.70; p < 0.01) (Fig. 2B) and females (r = 0.43; p < 0.01) (Fig. 4B) and a significant correlation between MV and CMJ with arms for males (r = 0.55; p < 0.01) (Fig. 2C) and females (r = 0.29; p < 0.05) (Fig. 4C).

## Discussion

The present study aimed to determine the relationship between vertical jump and muscle volume based on sex and age. We hypothesized that the differences in vertical jumping performance between sexes could be explained by differences in muscle volume.

The present study showed that muscle volume was different across age groups. The 20- to 22-year-old group presented higher muscle volume than the 9- to 10-year-old and 14- to 15-year-old groups. SJ, CMJ and CMJ arm height increased significantly with age for both males and females. In the 14- to 15-year-old age group, males performed significantly better than females. These differences persisted when performances were normalized to the length of the lower limb. Even after normalization to muscle volume, males exhibited better performance when compared to females. This difference persisted only for the 20–22-year-old group.

Concerning muscle volume, the results showed higher values among male participants than among females in the 9- to 10-year-old group. The results also showed that anthropometric variables (i.e., standing and sitting height, leg length, body mass) increased significantly with age for males and females. This finding could be explained by the effects of growth and maturity processes [38]. In fact, it was reported that during pubertal development, interactions between growth hormone, sex steroid hormones (i.e., oestrogens and androgens) and the production of insulin-like growth Factor I (IGF-I) induced changes in body composition and shape, including alterations in the relative proportions of water, muscle, fat and bone [38, 39]. Additionally, compared to males, females in the 14- to 15-year-old group had a higher body fat percentage (~ 49.36%), while males had a higher muscle volume (~ 32.38%). Our results are similar to those reported by Wells [2], who found that hormonal differences observed at puberty led to a significant increase in body fat percentage among girls and a significant increase in muscle mass among males. These physiological changes are associated with puberty, including the increase in testosterone levels, thereby inducing enlargement and differentiation of muscle fibres in boys compared to what is observed in girls, especially for fast-twitch fibres [40].

Moreover, our results showed that height (7.29%) and body fat percentage (16.30%) were higher in males than females during adulthood. These results are consistent with the findings of Shepard [1] and Withers et al. [41], who found that males are ≈ 13 cm larger, ≈ 14 to 18 kg heavier and have ≈ 10 to 15% less body fat than females [1, 41]. According to Shepard [1], size and body mass differences are two of the principal factors responsible for a 20% decrease in females' muscle power compared to males. Moreover, Withers et al. [41] showed that sex differences could be attributed to the lower muscle power observed among female participants. This study found that males had higher muscle volume (31.76%) and lower limb lengths (4.34%) than females. The current findings on age-related effects showed significant differences in males and females between the 9- to 10-year old and 14- to 15-year-old groups and between the 14- to 15-year-old and 20- to 22-year-old age groups, which may be a result of the simultaneous processes of growth and maturity. Considering jumping performance, a significant increase was shown with age for both sexes. The data showed that 14- to 15-year-old males jumped higher than females in the same age group. The results of this study are consistent with other research that emphasized this difference for children. [27, 42] and adults [43]. In comparison to the SJ, the CMJ height was higher for both males and females. It is generally known that jumping and other bounding movements can be improved by making a countermovement [44]. In the CMJ test, the greatest height reached could be explained by the active state initiated during the preparatory countermovement; in contrast, in the SJ, the countermovement is inevitably developed during the propulsion phase, so that the muscles can produce more force and work during shortening [44]. In this study, the maximum jump height difference between the CMJ and SJ was 2–3 cm, which is similar to the difference found by Bobbert and Casius (2–4 cm) [44]. The vertical jump height depends on many factors, such as the coordinated transfer of energy from the proximal to the distal joints [45]. The present study showed that females aged 9 to 10 years old performed almost equally as well as males in the SJ and CMJ, despite their lower muscle volume. According to Shepard [1], the difference in muscle volume observed between males and females aged 9 to 10 years old can mainly be attributed to educational and cultural factors that offer more frequent activities for males than females. For the 14- to 15-year-old age group, males exhibited significantly better performances in vertical jumps (SJ: 13.93%, CMJ: 24.88% and CMJ arms: 27.03%) than females. The differences in the CMJ height were maintained after normalized for the length of the lower limbs (14.29%), and the differences in the SJ height were maintained after adjusting for the lower limb muscle volume (11.90%). The difference in vertical jump performances between the sexes could be attributed to changes in body composition, particularly an increase in the percentage of muscle fibres with the increase in leg length and leg muscle volume among boys after the age of 13 years [27]. A significant sex difference was found in jumping performance in this study. Adult males jumped higher than females. This was reported previously by Taylor et al. [46]. This result resembles those of earlier studies that have been reported [47, 48], which showed that in comparison to females, males exhibited higher eccentric and concentric strength and power as well as greater peak power during the concentric phase of CMJ. Similarly, Kacem et al. [49] reported interesting results, showing that males are 30.4% more efficient than females in the five-jump test. Excess body fat for female has a disadvantageous effect on vertical jumps performances. These results are in agreement with those reported by Ben Mansour et al. [50], the persistence of sex differences after weighting of male students indicates that body fat is responsible for 30 to 70% of the observed differences between sexes performances and power outcomes during jump tests. Furthermore, Mayhew et al. [51] suggested that anaerobic power is linked to anthropometric dimensions and muscle power for both sexes. Even normalization to lower limb muscle volume in males elicited better performance than that in females. This difference persisted only for the 20-to 22-year-old age group. Our study showed that the differences between males and females in jumping performance persisted when performances were normalized for lower limb length. Vertical jump performances were significantly better among male participants than among females. This difference was mainly manifested among individuals aged 14–15 years old; the difference exceeded 50% between the ages of 20 and 22 years old. Sex differences persisted only for the 20- to 22-year-old group when performance was related to muscle volume. Moreover, our results indicate that the correlations between muscle volume and vertical jump height is stronger among males than among females (r > 0.50, p < 0.01). According to Hautier et al. [52], there is a significant correlation between the optimal pedalling frequency and the vastus lateralis muscle fibre composition. Regarding the differences between the sexes, it has also been suggested that the increase in testosterone levels in males induces a selective hypertrophy of type II muscle fibres [53]. According to our hypothesis, muscle volume could be considered one of the major determining factors in sex differences in vertical jumping performance. According to the results, muscle volume is a significant explanatory variable for jumping performance when sex is considered. Consequently, muscle volume can be proposed as a major factor that positively affects males' jump performance compared to females. There are some limitations to this study that must be acknowledged. The magnitude of the correlations may have been impacted by the relatively small sample sizes for each sex across the three age groups. Additionally, the traditional anthropometric method might have had an impact on the real values to estimate muscle volume. In other studies, proton magnetic resonance imaging (1H-MRI), dual-energy X-ray absorptiometry (DEXA), and quantitative computed tomography have been documented to provide accurate measurements of skeletal muscle volume [54, 55]. However, these methods are both complex and expensive, requiring sophisticated equipment in a purpose-designed setting, and are not widely available. Jumping performance is underpinned by several morphological and physiological factors, most notably muscle strength, body composition and power capabilities [56, 57].

## Conclusion

The results of this study indicated that muscle volume differed across age groups. For both sexes, the vertical jump height increased significantly with age. Male participants exhibited better performances than female participants in the vertical jump across all age groups. These differences were maintained when performances were normalized to the lower limbs 'length. Moreover, after normalization to lower limb muscle volume, sex differences were found only for the 20–22-year-old group. It can be concluded that muscle volume is a major influencing factor in sex differences in vertical jumping performance.

## Supplementary Information

**Additional file 1.** Anthropometric characteristics and Performance of vertical jump tests for all participants.

## Data Availability

The datasets used and/or analyzed during the current study are available from the corresponding author.
